# Global chromatin fibre compaction in response to DNA damage

**DOI:** 10.1016/j.bbrc.2011.10.021

**Published:** 2011-11-04

**Authors:** Charlotte Hamilton, Richard L. Hayward, Nick Gilbert

**Affiliations:** a Institute of Genetics and Molecular Medicine, The University of Edinburgh, Edinburgh EH4 2XR, UK; b Breakthrough Research Unit, The University of Edinburgh, Edinburgh EH4 2XR, UK

**Keywords:** NCS, neocarzinostatin, DSB, double strand break, MNase, micrococcal nuclease, DNA double strand breaks, Chromatin, Histones

## Abstract

DNA is protected by packaging it into higher order chromatin fibres, but this can impede nuclear processes like DNA repair. Despite considerable research into the factors required for signalling and repairing DNA damage, it is unclear if there are concomitant changes in global chromatin fibre structure. In human cells DNA double strand break (DSB) formation triggers a signalling cascade resulting in H2AX phosphorylation (γH2AX), the rapid recruitment of chromatin associated proteins and the subsequent repair of damaged sites. KAP1 is a transcriptional corepressor and in HCT116 cells we found that after DSB formation by chemicals or ionising radiation there was a wave of, predominantly ATM dependent, KAP1 phosphorylation. Both KAP1 and phosphorylated KAP1 were readily extracted from cells indicating they do not have a structural role and γH2AX was extracted in soluble chromatin indicating that sites of damage are not attached to an underlying structural matrix. After DSB formation we did not find a concomitant change in the sensitivity of chromatin fibres to micrococcal nuclease digestion. Therefore to directly investigate higher order chromatin fibre structures we used a biophysical sedimentation technique based on sucrose gradient centrifugation to compare the conformation of chromatin fibres isolated from cells before and after DNA DSB formation. After damage we found global chromatin fibre compaction, accompanied by rapid linker histone dephosphorylation, consistent with fibres being more regularly folded or fibre deformation being stabilized by linker histones. We suggest that following DSB formation, although there is localised chromatin unfolding to facilitate repair, the bulk genome becomes rapidly compacted protecting cells from further damage.

## Highlights

► Robust KAP1 phosphorylation in response to DNA damage in HCT116 cells. ► DNA repair foci are found in soluble chromatin. ► Biophysical analysis reveals global chromatin fibre compaction after DNA damage. ► DNA damage is accompanied by rapid linker histone dephosphorylation.

## 1 Introduction

In mammalian cells DNA is packaged with histone proteins into nucleosomes which fold to form a 30-nm diameter chromatin fibre that are further packaged into large scale chromatin structure [1]. Chromatin both protects the DNA from damage but also provides a regulated environment for nuclear processes such as transcription, replication and DNA repair. DNA double strand breaks (DSBs) lead to chromosomal fragmentation and cause genomic rearrangements if not repaired [2]. To maintain genome stability, cells possess a surveillance system called the DNA damage response (DDR) which recognises and repairs DNA damage and initiates check point events that control G1/S progression [3]. After the induction of DSBs either by ionising radiation or chemically by agents such as neocarzinostatin (NCS) the damage is sensed by a process that remains controversial but activates a signalling pathway via the PI3-kinase related protein kinases (PIKKs) ATM, ATR and DNA-PK. ATM becomes autophosphorylated triggering a signalling cascade promoting the phosphorylation of H2AX marking 1 Mb domains around the sites of damage, binding of the MDC1 adapter protein and recruitment of 53BP1.

In areas surrounding DSBs there is chromatin movement, cytologically visible localised expansion, and by LM/ESI (light microscopy/electron spectroscopic imaging), chromatin with the appearance of 10-nm fibres have been seen within repair foci [4]. There are also changes in chromatin associated proteins in response to DNA DSBs including the recruitment of HP1 and KAP1 to sites of damage in a p150CAF-1 dependent manner [5] that may function to reorganise chromatin. Furthermore loss of HP1 results in high sensitivity to DNA DSBs [6], possibly by making the chromatin more accessible to damage. KAP1 is an abundant nuclear protein that promotes the formation of transcriptionally repressed heterochromatin-like structures. In response to damage, KAP1 is phosphorylated in an ATM-dependent manner at damage sites, from where it spreads throughout the nucleus [7,8]. Heterochromatin provides a barrier for DNA repair and KAP1 phosphorylation is required for repairing damage in heterochromatin, but depletion of HP1 proteins alleviates the need for pKAP1 at heterochromatin [9,10] suggesting they are both involved in modulating chromatin structure.

Changes in global chromatin organisation are seen in a number of physiological situations including the initiation of apoptosis, mitosis and the formation of facultative heterochromatin in the final stages of differentiation in nucleated erythrocytes. However, it is important to distinguish between changes in chromatin structure that occur at the level of the 30-nm chromatin fibre and higher levels of chromatin organisation. During facultative heterochromatin formation in chicken erythrocytes the variant linker histone H5 becomes dephosphorylated [11,12] promoting chromatin compaction by increasing electrostatic interactions [13] between histone proteins and the DNA. Ionising radiation also triggers the rapid but transient dephosphorylation of linker histones in Jurkat and RKO cells in an ATM dependent manner [14]. In contrast, during mitosis histone H1 becomes phosphorylated, possibly relaxing the chromatin fibre to enable remodelling to a subsequently more compact structure.

In response to DNA DSBs chromatin decondensation and a global increase in chromatin accessibility have been reported [15–17] and by LM/ESI DSBs cause a global decrease in chromatin density [4]. Furthermore, KAP1 depletion, or mimicking constitutive phosphorylation of this protein has been suggested to lead to constitutive global chromatin “relaxation” that might facilitate DNA damage repair [7]. However, global chromatin fibre relaxation would potentially render the DNA at greater risk of damage. To investigate whether DNA damage repair is accompanied by a global reconfiguration of fundamental chromatin fibres we have induced DNA damage and investigated the chromatin structure using nuclease sensitivity assays and a biophysical sedimentation approach that measures chromatin fibre compaction. Although we find a rapid change in KAP1 and H2AX phosphorylation we do not find a change in nuclease sensitivity of the chromatin fibres. However, we find that in response to DNA DSBs the chromatin fibre adopts a more compact structure, consistent with fibres being more regularly folded or fibre deformations being stabilized. This global compaction of chromatin fibres could therefore protect the genome from subsequent damage.

## 2 Materials and methods

### 2.1 Cell lines and reagents

Human HCT116 and U2OS cells were maintained in Dulbecco’s modified Eagle’s medium supplemented with 10% foetal calf serum (FCS). All reagents were purchased from Sigma. The specific ATM inhibitor KU55933 and the specific DNA-PK inhibitor NU7441 were gifts from KUDOS. DNA damage was either introduced by treatment with the radiomimetic agent neocarzinostatin (NCS) (50–200 ng/ml) or by γ-irradiation (2–30 Gy) from a Co60 source. For colony forming assays cells were γ-irradiated or treated with NCS and plated at different densities. Cells were grown for 9 days, stained using 0.4% sulforhodamine B and the colonies counted.

### 2.2 Western blotting

Protein was fractionated on a 10% or 12% SDS polyacrylamide gel, transferred to Hybond P, and membranes were probed with antibodies that detect the following: KAP1 (1:1000; Bethyl Laboratories), pS824 KAP1 (1:1000; Bethyl Laboratories), H2AX (1:1000; 07-627, Millipore), γH2AX (1:500; 05-636, Millipore), HP1α (1:500; MAB3446, Millipore), HP1β (1:500; MAB3448; Millipore), hyperphosphorylated H1 (1:500; 06-597, Millipore), and glyceraldehyde-3-phosphate dehydrogenase (GAPDH; 1:2000, Abcam). Detection was performed by ECL.

### 2.3 Immunofluorescence

Human U2OS cells were grown on slides and fixed using 4% pFa in PBS [18]. The cells were permeabilized using Triton X-100 in PBS and were sequentially incubated with pS824 KAP (1:100), γH2AX (1:100) and secondary antibodies (Jackson Laboratories).

### 2.4 Preparation and fractionation of cells, nuclei and chromatin

To fractionate cells into supernatant and pellet fractions, cells were scrapped into gentle lysis buffer (PBS supplemented with 0.5% TX-100, 5 mM EDTA, 200 μM PMSF) and incubated for 30 min with mixing. The samples were centrifuged at max speed for 5 min. Supernatant and pellets were taken and resuspended in equivalent volumes 2× SDS sample buffer, boiled and sonicated. Nuclei were prepared as described [18]. For micrococcal nuclease (MNase) sensitivity digests, the nuclei concentration was adjusted to 4 A260 in nuclei buffer R. About 50 U/ml MNase (Worthington) was added, and aliquots were removed into stop buffer (2% SDS, 200 μg/ml proteinase K, and 10 mM EDTA) at various time intervals. Purified DNAs were fractionated on a 1% agarose gel in Tris–borate buffer in the presence of EtBr. Soluble chromatin was prepared and fractionated on 6–40% isokinetic sucrose gradients in TEEP80 buffer (10 mM Tris–HCl pH 8, 1 mM EDTA, 1 mM EGTA, 80 mM NaCl, 250 μM PMSF) as described previously [19,20]. Gel images were analysed using the Aida software package version 4.22 (Raytek).

## 3 Results and discussion

### 3.1 Chromatin modifications in response to DNA DSBs

**Fig. 1:**
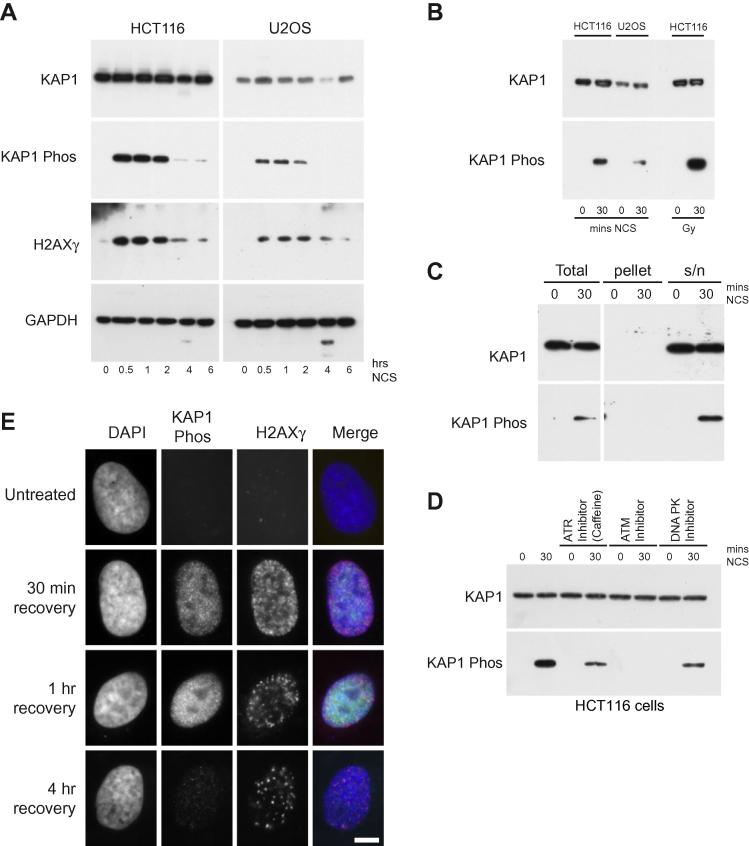
KAP1 phosphorylation in HCT116 cells in response to DNA damage. (A) HCT116 and U2OS cells were treated with 200 ng/ml neocarzinostatin (NCS). At specific time points (until 6 h) protein was extracted and Western blotted for KAP1, pS824 KAP1, γH2AX. (B) HCT116 cells were exposed to 30 Gy γ-radiation, protein was isolated and Western blotted for KAP1 and pS824 KAP1. (C) HCT116 cells were treated with NCS, extracted with 0.5% Triton X-100 to give soluble and insoluble fractions. Protein samples were recovered, Western blotted and probed for KAP1 and pS824 KAP1. (D) To investigate the predominant phosphatidyl inositol 3′ kinase-related kinases (PIKK) responsible for phosphorylating S824 KAP1 in HCT116 cells, cells were treated with either 1 mM caffeine (ATR inhibitor) for 2.5 h, 10 μM KU55933 (ATM inhibitor) or 1 μM NU7441 (DNA-PK inhibitor) for 1 h and then exposed to NCS for 30 min. Proteins were extracted and Western blotted and probed for KAP1 and pS824 KAP1. (E) Formation of DNA damage foci in U2OS cells. Cells grown on slides were treated with 200 ng/ml NCS for 30 min, paraformaldehyde fixed and probed for γH2AX (red) and pS824 KAP1 (green) and counterstained with 50 μg/ml DAPI. Scale bar is 5 μm.

Mammalian cells rapidly sense DNA damage and elicit a response. It has been suggested that part of this process is to trigger a global remodelling of chromatin structures to facilitate DNA damage repair. DNA strand breaks can be introduced by ionising radiation (e.g. γ-rays) or the radiomimetic agent neocarzinostatin (NCS). We have investigated the global effects of DNA DSBs in U2OS and HCT116 cells. Previously it has been shown that DNA damage promotes the rapid phosphorylation of KAP1 [7,8]. We confirmed this response in U2OS cells and in HCT116 cells. After treatment of cells with NCS there was a rapid phosphorylation of KAP1 which persisted for approximately 2 h before fading away (Fig. 1A). H2AX phosphorylation is one of the earliest events in the DNA damage response. After NCS treatment H2AX was rapidly phosphorylated and this mark persisted for up to 6 h. KAP1 phosphorylation was also induced by γ irradiation in HCT116 cells to an even greater extent, demonstrating these cells have a robust response to DNA DSBs (Fig. 1B). Furthermore, as KAP1 phosphorylation is stronger in HCT116 cells than U2OS cells it suggests there might be cell line specific differences. This could be due to an increased level of KAP1 in HCT116 cells or that these cells either have increased levels of DNA damage kinases or that these cells are particularly efficient at sensing DNA DSBs. To investigate whether the associated KAP1 with chromatin changes upon phosphorylation, cells were exposed to NCS and extracted in gentle lysis buffer. Soluble proteins are readily extracted whilst histones and tightly associated proteins remain in the pellet. KAP1 is phosphorylated after DNA damage but pKAP1 remains in the soluble fraction (Fig. 1C) suggesting that KAP1 has a role in signalling DNA damage response rather than becoming structurally associated with chromatin or an underlying matrix.

Previously KAP1 phosphorylation was shown to be dependent on ATM signalling [7], but other studies [8] show that KAP1 phosphorylation occurs in ATM deficient cells, demonstrating that KAP1 might be redundantly targeted for phosphorylation by all three nuclear PIKK family members. To investigate which kinases operated in HCT116 cells to phosphorylate KAP1, damaged cells were treated with PIKK inhibitors: Caffeine (ATR), KU55933 (ATM) or NU7441 (DNA PK). The ATM inhibitor strongly blocked KAP1 phosphorylation indicating that this pathway is required in HCT116 cells for KAP1 phosphorylation (Fig. 1D). Surprisingly there was also a reduction in KAP1 phosphorylation upon treatment with both the ATR and DNA-PK suggesting that these two pathways are also involved in KAP1 phosphorylation but are secondary to ATM.

To visualise the formation of DNA damage lesions U2OS cells were treated with 200 ng/ml NCS and stained for KAP1 phosphorylation and γH2AX over a 4 h period. Small KAP1 phosphorylation foci were visible after 30 min, increasing to 1 h, but were virtually undetectable at 4 h (Fig. 1E). In contrast, γH2AX foci continued to be resolved at 4 h giving strong punctuate foci. γH2AX spreads forming large domains [21] which might be important for localising chromatin remodellers to repair sites of DNA damage. Previously DNA damage has been shown to promote the local decondensation of chromatin to facilitate repair. Over this time course, we were unable to detect any gross changes by DAPI staining of the nucleus and there was no apparent alteration in nuclear volume.

### 3.2 Chromatin structure changes in response to DNA DSBs

**Fig. 2:**
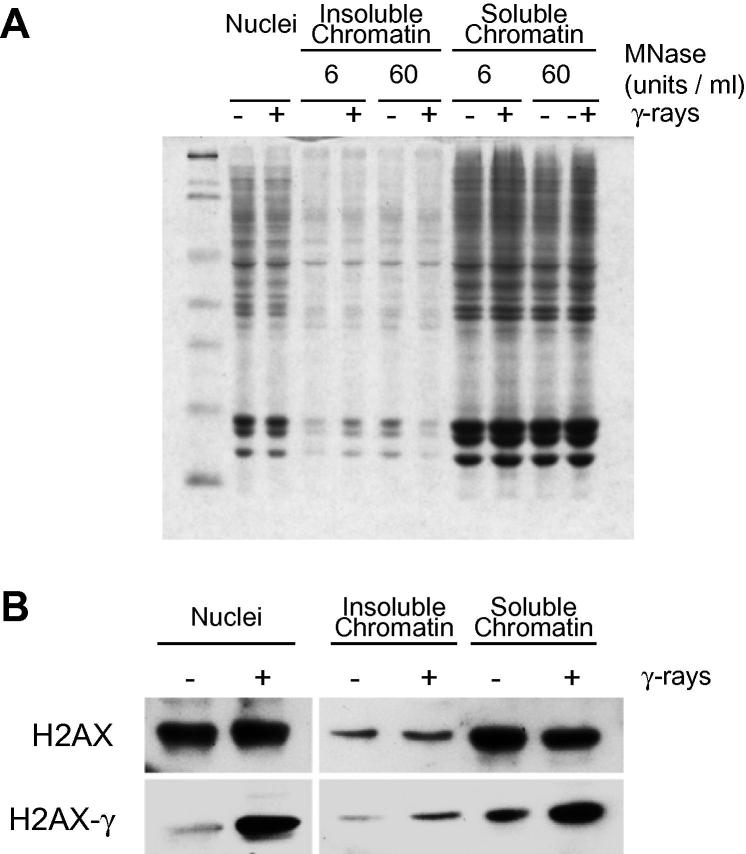
Chromatin association of H2AX and γH2AX. HCT116 cells were irradiated (30 Gy), nuclei were purified, digested with micrococcal nuclease (6 or 60 units/ml) and soluble and insoluble chromatins were extracted. (A) Coomassie stained gel of HCT116 nuclei, soluble chromatin proteins and insoluble chromatin proteins. (B) Western blot analysis of H2AX and γH2AX in nuclei, soluble and insoluble chromatin.

To investigate whether DNA damage was associated with a redistribution of chromatin proteins we examined whether γH2AX was associated with soluble chromatin or if after damage it becomes attached to an underlying nuclear matrix. Cells were treated with γ-rays, nuclei were prepared and briefly digested with MNase. After centrifuging the supernatant (SN1) was discarded and the pellet was resuspended in TEEP20 buffer (10 mM Tris–HCl pH 8, 1 mM EDTA, 1 mM EGTA, 20 mM NaCl, 250 μM PMSF) overnight. The following day the sample was centrifuged and the supernatant (SN2) and pellet (P) were recovered. Most histone proteins are extracted as soluble chromatin in SN2 and the protein distribution is similar between untreated and irradiated cells (Fig. 2A). H2AX is predominantly found in soluble chromatin (Fig. 2B) and its distribution does not appear to change after DNA damage. γH2AX is also extracted in soluble chromatin indicating that sites of DNA repair do not become associated with insoluble nuclear structures. Furthermore, although γH2AX has been shown to spread to form large domains [21] these structures are still extractable.

**Fig. 3:**
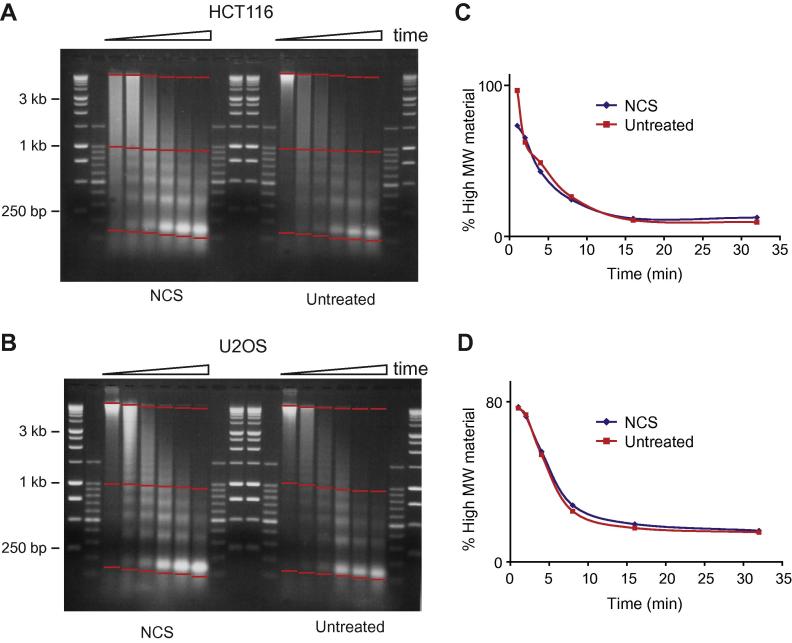
Nuclease sensitivity analysis after DNA damage. HCT116 and U2OS cells were treated with 200 ng/ml NCS for 30 min. Nuclei were purified and digested with micrococcal nuclease (50 units/ml) for different amounts of time (1–32 min). (A and B) DNA was isolated, purified and fractionated on a 1% agarose gel. Markers are 1 Kb and 100 bp ladder. (C and D) Quantification and analysis of gels in A and B showing the % loss of high molecular weight material (calculated as signal between the top two red lines divided by the signal between the top and bottom red lines).

Previous studies have suggested that DNA damage is accompanied by a global change in chromatin structure [7,15,17,22]. A simple approach to assess global chromatin structure is to digest nuclei with micrococcal nuclease [23]. If the chromatin is more unfolded it would be expected to digest more rapidly. We initially investigated chromatin compaction by treating cells with NCS which triggers, KAP1 phosphorylation, H2AX phosphorylation (Fig. 1) but giving a low level of cell death (data not shown). After treatment, nuclei were prepared from cells and digested with MNase to probe for changes in chromatin accessibility. After digestion, DNA was purified, fractionated by agarose gel electrophoresis (Fig. 3A and B) and the loss of high MW material was determined (Fig. 3C and D). This analysis can be prone to artefacts due to the rapid digestion of damaged nuclei or chromatin giving rise to excessive mono-nucleosomes. So measuring the % loss of high MW material is a more robust strategy and corrects for sample loading. Both HCT116 and U2OS cells are similarly sensitive to MNase digestion in the presence or absence of DNA damage in contrast to what has been found previously [7,15,17]. We were also unable to observe a change in nucleosome repeat length as has been previously documented [7]. This data suggests that although cells detect damage and elicit a response, this is local to the sites of damage and by this assay there is no global affect on nuclease accessibility. Previously, we have shown that major satellite is more sensitive to nuclease to bulk chromatin in mouse cells [23] demonstrating this technique can differentiate between different chromatin structures. This assay is affected by how permeable the nuclear membrane is to the nucleases. It is therefore possible that in previous studies the nuclear membrane of cells exposed to DNA damage was more fragile than wild type cells giving the impression that the chromatin is more readily digested by nuclease. This is consistent with reports that nuclei expand in response to damage [4] which may strain the membrane enabling nuclease to more readily pass through and access the chromatin. In our analyses as the shapes of the digestion curves were very similar it indicates that both treated and untreated cells are being digested in a similar manner.

**Fig. 4:**
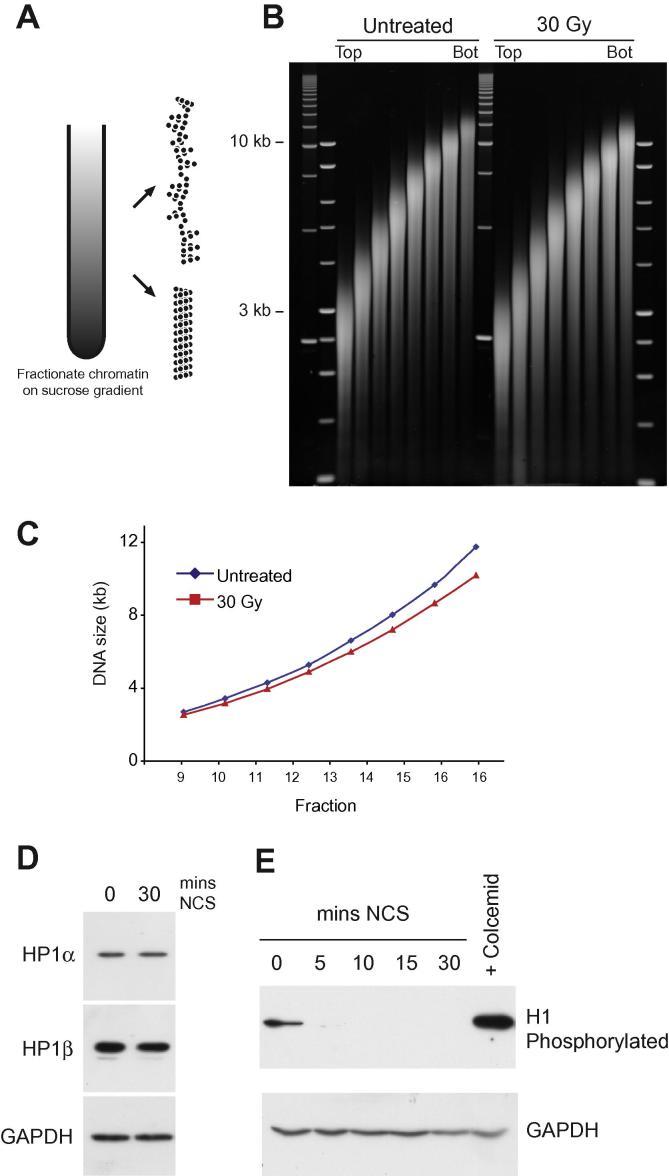
Chromatin fibre compaction in response to γ-irradiation. HCT116 cells were exposed to 30 Gy γ-irradiation and large soluble chromatin fragments were isolated under physiological conditions. (A) Soluble chromatin was sedimented on a 6–40% sucrose gradient in TEEP80 buffer and fractionated by upward displacement. (B) DNA was purified from individual fractions and analysed on a 0.7% agarose gel in TPE buffer. Markers are 2.5 Kb and 1 Kb ladders. (C) Quantification of chromatin sedimentation relating chromatin (DNA) size to sedimentation rate (faction number) showing that chromatin fragments isolated from γ-irradiated cells sediment more rapidly than chromatin isolated from untreated cells of the same size. (D) Western blot analysis of HP1α and HP1β in cells treated with 200 ng/ml NCS (30 min). E. Analysis of linker histone phosphorylation in HCT116 cells treated with NCS.

To further investigate whether there is a global change in chromatin structures in response to DNA damage we investigated the biophysical sedimentation properties of bulk chromatin fibres isolated from HCT116 cells exposed to ionising radiation. Previously, we have used this approach to demonstrate that satellite containing chromatin fibres have a compact structure compared to bulk chromatin fibres [19] and to map the distribution of open chromatin across the genome [20]. Essentially, this technique works by comparing the sedimentation properties of chromatin fibres isolated from treated and untreated cells under physiological conditions, that maintain the folding of the higher order chromatin fibres. Chromatin fibres of the same mass (size) will sediment in the same fraction in the sucrose gradient if they have the same structure. In contrast chromatin fibres of the same mass (size) but different structures will sediment differently (Fig. 4A). For example a compact chromatin fibre will sediment more rapidly than a chromatin fibre that is unfolded or interspersed with many disruption and previously we have used the sedimentation properties to model the chromatin fibre structure [19]. If DNA damage is accompanied by a decompaction of global chromatin fibres, we would expect to detect it.

Cells were exposed to γ-radiation (30 Gy) and nuclei were prepared from the cells and briefly digested with MNase (6 units/ml). Soluble chromatin was released from the nuclei under physiological conditions and was fractionated on a 6–40% isokinetic sucrose gradient in TEEP80 buffer. After sedimentation, individual fractions were isolated and analysed by agarose gel electrophoresis (Fig. 4B). Analysis of the sedimentation of the bulk chromatin fibres in each fraction showed that chromatin from untreated and DNA damage cells sediment differently (Fig. 4C). Chromatin fibres isolated from cells exposed to γ-radiation sediment more rapidly than fibres from untreated cells; this is most easily conceptualised by comparing the sedimentation rate (fraction number) of chromatins of the same size. Previously we have modelled the structure of chromatin fibres and suggested that chromatin fibres are dynamic structures that are interspersed with discontinuities [19]. This data indicates that after DNA damage the chromatin fibres are more regularly folded (fewer disruptions) or fibre deformations are stabilized resulting in the fibres having a more compact structure.

As there is a change in chromatin fibre sedimentation but no difference in nuclease sensitivity (Fig. 3) it raises the possibility that these two techniques are examining different levels of chromatin organisation. Sucrose sedimentation is directly analysing the sedimentation properties of the chromatin fibre. In contrast nuclease sensitivity is examining the rate at which the nuclease can attack the chromatin. This might be affected by membrane permeability and chromatin density which could be a reflection of higher levels of chromatin organisation. Chromatin density is reduced after damage [4] and this might allow access to the nuclease explaining previous results [7,17]. However this is contrary to the results we present here (Fig. 3) as we find no difference in nuclease sensitivity so could be a reflection of how the nuclei are prepared. In our experiments the nuclei were prepared in a very gentle manner whereas other approaches might influence the nuclei architecture revealing a difference in nuclease sensitivity, that is not a direct reflection of chromatin compaction.

As our data suggests that after DNA damage the chromatin fibre has shifted to a more rigid structure (Fig. 4), we examined which factors might be responsible. HP1 is an adapter protein that can bind a number of other proteins through its chromoshadow domain. HP1α accumulates at both euchromatin and heterochromatin after DNA damage in a p150CAF-1 dependent manner [5]. Depletion of p150CAF-1 or HP1α leads to defects in the DDR and cell survival. It also plays a role in DNA repair by homologous recombination, possibly by promoting end resection. To address whether a change in global compaction can be attributed an alteration in HP1 levels we Western blotted for HP1α and HP1β after DNA damage. As there was no change in the level of the HP1s in cells (Fig. 4D) we investigated what other proteins might be responsible for the change in global chromatin fibre compaction.

One of the key determinants of chromatin fibre compaction is linker histone H1 [24]. Histone H1 is highly dynamic and has long N and C terminal tails that can stabilize chromatin fibre interactions. These interactions are generally electrostatic in nature and are affected by linker histone phosphorylation. DNA damage has been shown to induce a rapid reduction in linker histone phosphorylation [14]. We treated HCT116 cells with NCS and then Western blotted and probed for hyper phosphorylated H1 (Fig. 4E). After DNA damage there was a rapid loss in linker histone phosphorylation. As loss of linker histone phosphorylation is seen in the compaction of chromatin in the maturation of chicken erythrocytes [11,25] we suggest that a change in linker histone phosphorylation in HCT116 cells after DNA damage will compact the chromatin fibre.

Our data demonstrates that DNA damage in HCT116 cells triggers a cellular response leading to the phosphorylation of the chromatin associated proteins KAP1 and H2AX. In addition there is a rapid loss of linker histone phosphorylation. Although DNA damage requires a localised decompaction of chromatin fibres for repair our data indicates that it is accompanied by genome-wide compaction of bulk chromatin fibres which we suggest is caused by linker histone phosphorylation. Chromatin fibre compaction has been shown to limit the effect of DNA damage and this response might protect cells from further damage whilst they are repairing other damaged regions.
